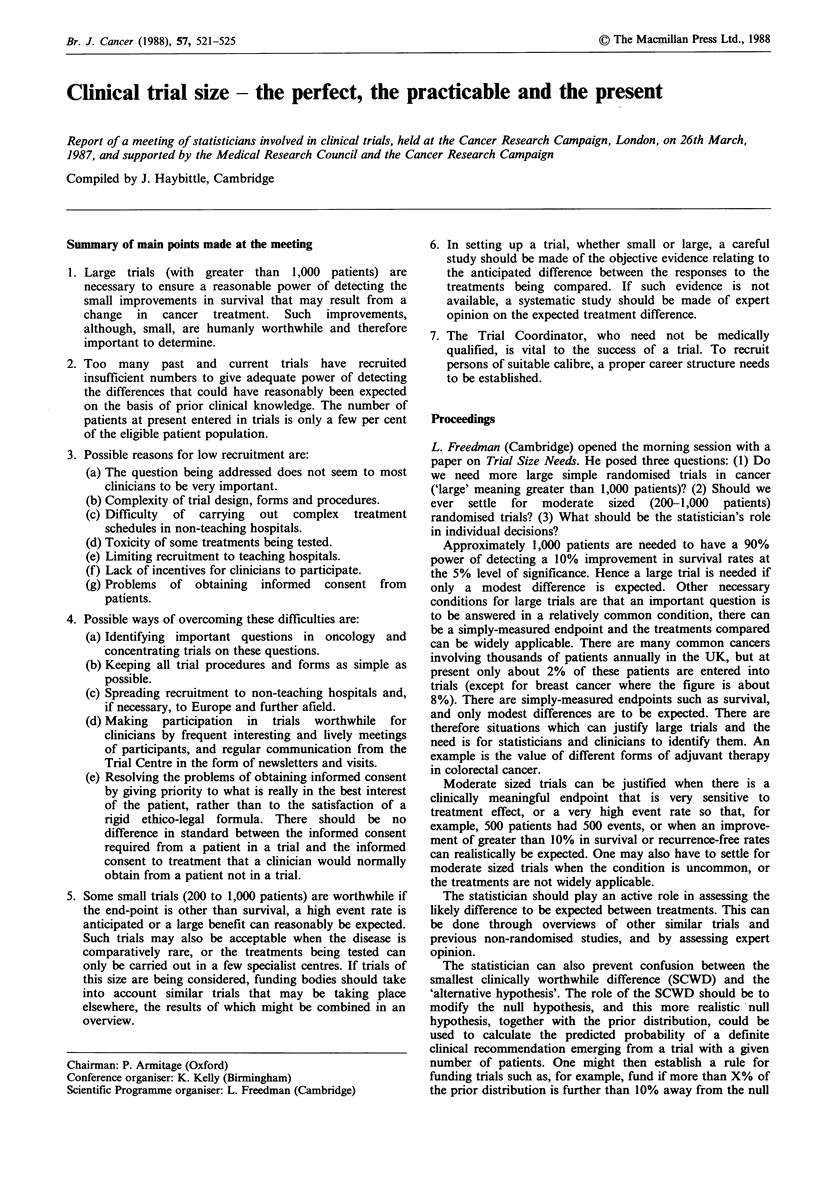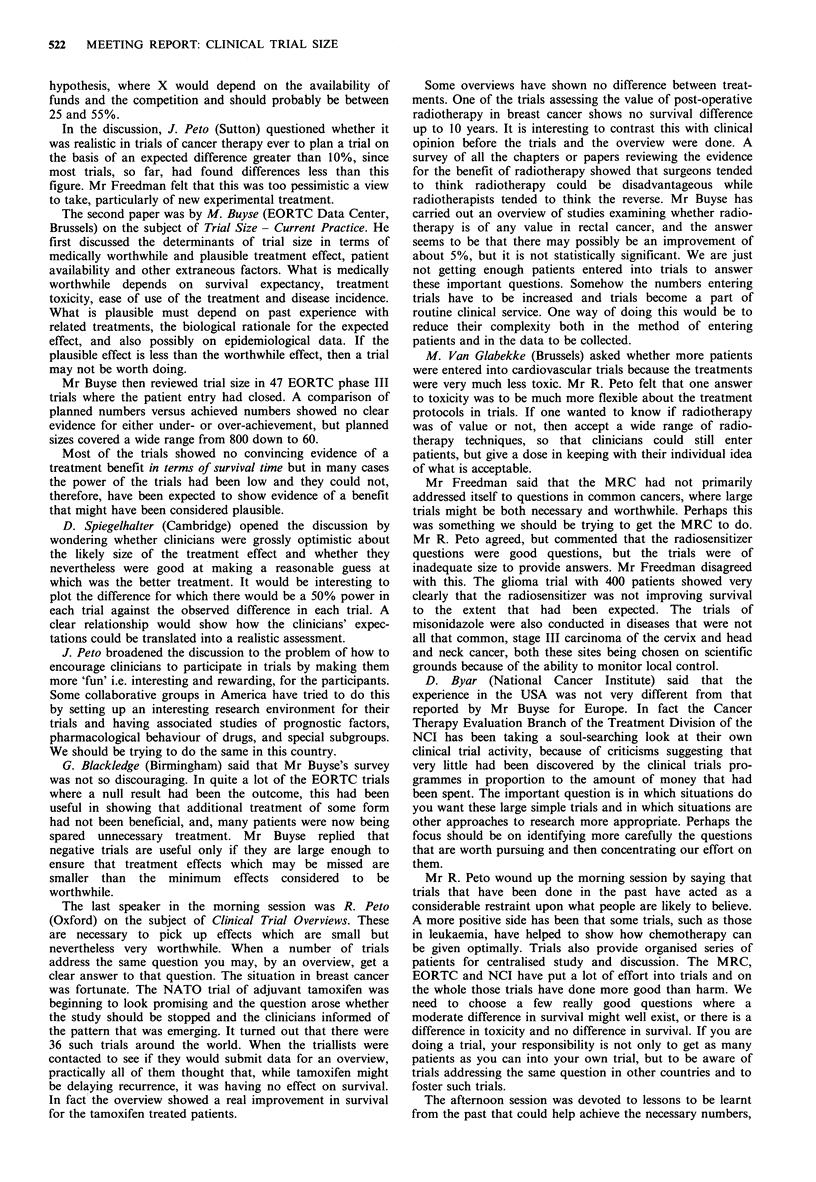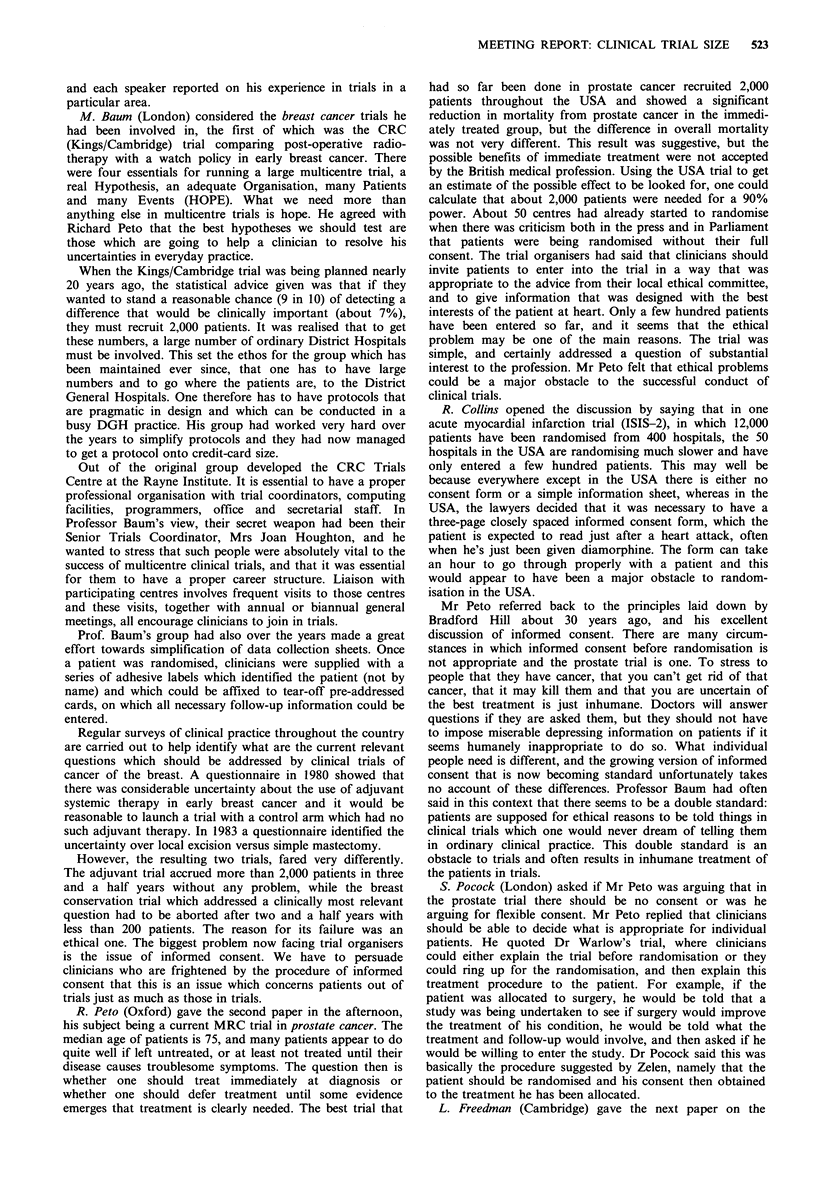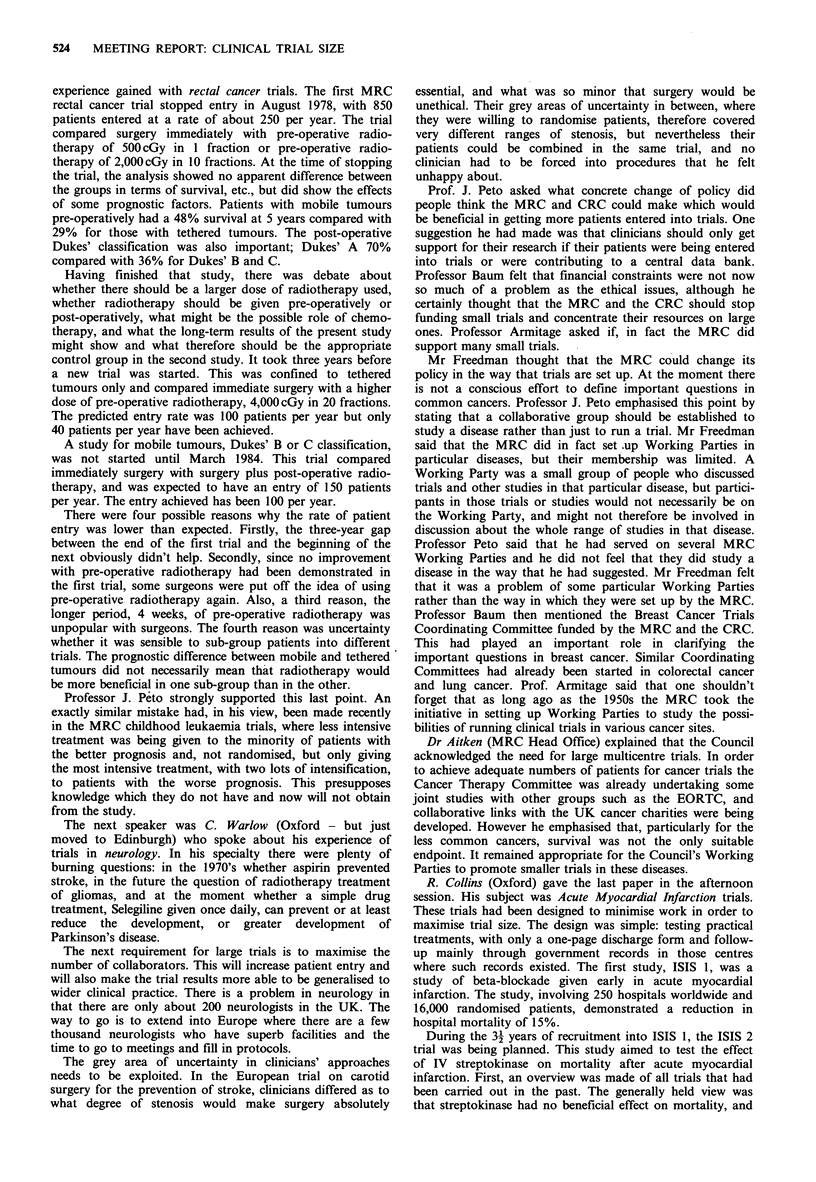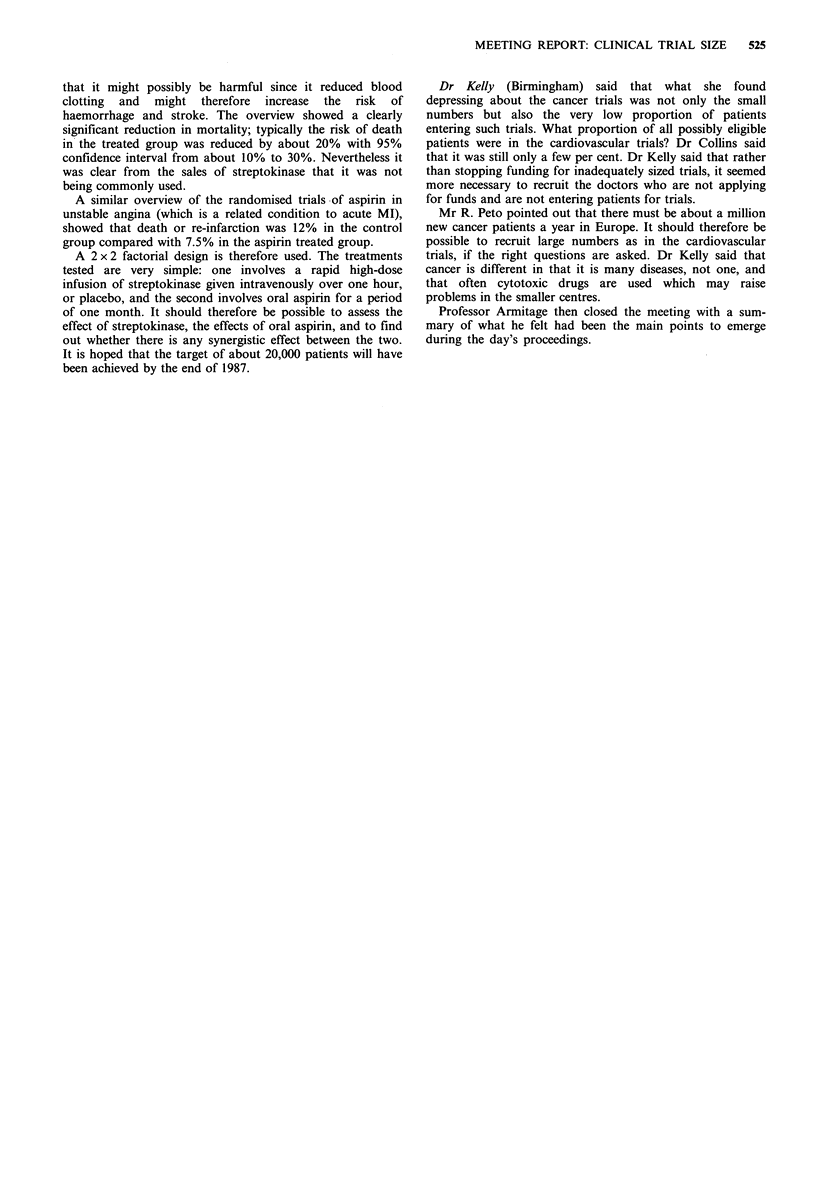# Clinical trial size - the perfect, the practicable and the present

**Published:** 1988-05

**Authors:** 


Br. J. Cancer (1988), 57, 521–525 © The Macmillan Press Ltd., 1988

### Clinical trial size – the perfect, the practicable and the present

*Report of a meeting of statisticians involved in clinical trials, held at the Cancer Research Campaign, London, on 26th March, 1987, and supported by the Medical Research Council and the Cancer Research Campaign*

Compiled by J. Haybittle, Cambridge

**Summary of main points made at the meeting**

1. Large trials (with greater than 1,000 patients) are necessary to ensure a reasonable power of detecting the small improvements in survival that may result from a change in cancer treatment. Such improvements, although, small, are humanly worthwhile and therefore important to determine.
2. Too many past and current trials have recruited insufficient numbers to give adequate power of detecting the differences that could have reasonably been expected on the basis of prior clinical knowledge. The number of patients at present entered in trials is only a few per cent of the eligible patient population.
3. Possible reasons for low recruitment are:
   (a) The question being addressed does not seem to most clinicians to be very important.
   (b) Complexity of trial design, forms and procedures.
   (c) Difficulty of carrying out complex treatment schedules in non-teaching hospitals.
   (d) Toxicity of some treatments being tested.
   (e) Limiting recruitment to teaching hospitals.
   (f) Lack of incentives for clinicians to participate.
   (g) Problems of obtaining informed consent from patients.
4. Possible ways of overcoming these difficulties are:
   (a) Identifying important questions in oncology and concentrating trials on these questions.
   (b) Keeping all trial procedures and forms as simple as possible.
   (c) Spreading recruitment to non-teaching hospitals and, if necessary, to Europe and further afield.
   (d) Making participation in trials worthwhile for clinicians by frequent interesting and lively meetings of participants, and regular communication from the Trial Centre in the form of newsletters and visits.
   (e) Resolving the problems of obtaining informed consent by giving priority to what is really in the best interest of the patient, rather than to the satisfaction of a rigid ethico-legal formula. There should be no difference in standard between the informed consent required from a patient in a trial and the informed consent to treatment that a clinician would normally obtain from a patient not in a trial.
5. Some small trials (200 to 1,000 patients) are worthwhile if the end-point is other than survival, a high event rate is anticipated or a large benefit can reasonably be expected. Such trials may also be acceptable when the disease is comparatively rare, or the treatments being tested can only be carried out in a few specialist centres. If trials of this size are being considered, funding bodies should take into account similar trials that may be taking place elsewhere, the results of which might be combined in an overview.
6. In setting up a trial, whether small or large, a careful study should be made of the objective evidence relating to the anticipated difference between the responses to the treatments being compared. If such evidence is not available, a systematic study should be made of expert opinion on the expected treatment difference.
7. The Trial Coordinator, who need not be medically qualified, is vital to the success of a trial. To recruit persons of suitable calibre, a proper career structure needs to be established.

**Proceedings**

*L. Freedman* (Cambridge) opened the morning session with a paper on *Trial Size Needs*. He posed three questions: (1) Do we need more large simple randomised trials in cancer ('large' meaning greater than 1,000 patients)? (2) Should we ever settle for moderate sized (200–1,000 patients) randomised trials? (3) What should be the statistician's role in individual decisions?

Approximately 1,000 patients are needed to have a 90% power of detecting a 10% improvement in survival rates at the 5% level of significance. Hence a large trial is needed if only a modest difference is expected. Other necessary conditions for large trials are that an important question is to be answered in a relatively common condition, there can be a simply-measured endpoint and the treatments compared can be widely applicable. There are many common cancers involving thousands of patients annually in the UK, but at present only about 2% of these patients are entered into trials (except for breast cancer where the figure is about 8%). There are simply-measured endpoints such as survival, and only modest differences are to be expected. There are therefore situations which can justify large trials and the need is for statisticians and clinicians to identify them. An example is the value of different forms of adjuvant therapy in colorectal cancer.

Moderate sized trials can be justified when there is a clinically meaningful endpoint that is very sensitive to treatment effect, or a very high event rate so that, for example, 500 patients had 500 events, or when an improvement of greater than 10% in survival or recurrence-free rates can realistically be expected. One may also have to settle for moderate sized trials when the condition is uncommon, or the treatments are not widely applicable.

The statistician should play an active role in assessing the likely difference to be expected between treatments. This can be done through overviews of other similar trials and previous non-randomised studies, and by assessing expert opinion.

The statistician can also prevent confusion between the smallest clinically worthwhile difference (SCWD) and the 'alternative hypothesis'. The role of the SCWD should be to modify the null hypothesis, and this more realistic null hypothesis, together with the prior distribution, could be used to calculate the predicted probability of a definite clinical recommendation emerging from a trial with a given number of patients. One might then establish a rule for funding trials such as, for example, fund if more than X% of the prior distribution is further than 10% away from the null

Chairman: P. Armitage (Oxford)
Conference organiser: K. Kelly (Birmingham)
Scientific Programme organiser: L. Freedman (Cambridge)

hypothesis, where X would depend on the availability of funds and the competition and should probably be between 25 and 55%.

In the discussion, *J. Peto* (Sutton) questioned whether it was realistic in trials of cancer therapy ever to plan a trial on the basis of an expected difference greater than 10%, since most trials, so far, had found differences less than this figure. Mr Freedman felt that this was too pessimistic a view to take, particularly of new experimental treatment.

The second paper was by *M. Buyse* (EORTC Data Center, Brussels) on the subject of *Trial Size – Current Practice.* He first discussed the determinants of trial size in terms of medically worthwhile and plausible treatment effect, patient availability and other extraneous factors. What is medically worthwhile depends on survival expectancy, treatment toxicity, ease of use of the treatment and disease incidence. What is plausible must depend on past experience with related treatments, the biological rationale for the expected effect, and also possibly on epidemiological data. If the plausible effect is less than the worthwhile effect, then a trial may not be worth doing.

Mr Buyse then reviewed trial size in 47 EORTC phase III trials where the patient entry had closed. A comparison of planned numbers versus achieved numbers showed no clear evidence for either under- or over-achievement, but planned sizes covered a wide range from 800 down to 60.

Most of the trials showed no convincing evidence of a treatment benefit *in terms of survival time* but in many cases the power of the trials had been low and they could not, therefore, have been expected to show evidence of a benefit that might have been considered plausible.

*D. Spiegelhalter* (Cambridge) opened the discussion by wondering whether clinicians were grossly optimistic about the likely size of the treatment effect and whether they nevertheless were good at making a reasonable guess at which was the better treatment. It would be interesting to plot the difference for which there would be a 50% power in each trial against the observed difference in each trial. A clear relationship would show how the clinicians' expectations could be translated into a realistic assessment.

*J. Peto* broadened the discussion to the problem of how to encourage clinicians to participate in trials by making them more 'fun' i.e. interesting and rewarding, for the participants. Some collaborative groups in America have tried to do this by setting up an interesting research environment for their trials and having associated studies of prognostic factors, pharmacological behaviour of drugs, and special subgroups. We should be trying to do the same in this country.

*G. Blackledge* (Birmingham) said that Mr Buyse's survey was not so discouraging. In quite a lot of the EORTC trials where a null result had been the outcome, this had been useful in showing that additional treatment of some form had not been beneficial, and, many patients were now being spared unnecessary treatment. Mr Buyse replied that negative trials are useful only if they are large enough to ensure that treatment effects which may be missed are smaller than the minimum effects considered to be worthwhile.

The last speaker in the morning session was *R. Peto* (Oxford) on the subject of *Clinical Trial Overviews.* These are necessary to pick up effects which are small but nevertheless very worthwhile. When a number of trials address the same question you may, by an overview, get a clear answer to that question. The situation in breast cancer was fortunate. The NATO trial of adjuvant tamoxifen was beginning to look promising and the question arose whether the study should be stopped and the clinicians informed of the pattern that was emerging. It turned out that there were 36 such trials around the world. When the triallists were contacted to see if they would submit data for an overview, practically all of them thought that, while tamoxifen might be delaying recurrence, it was having no effect on survival. In fact the overview showed a real improvement in survival for the tamoxifen treated patients.

Some overviews have shown no difference between treatments. One of the trials assessing the value of post-operative radiotherapy in breast cancer shows no survival difference up to 10 years. It is interesting to contrast this with clinical opinion before the trials and the overview were done. A survey of all the chapters or papers reviewing the evidence for the benefit of radiotherapy showed that surgeons tended to think radiotherapy could be disadvantageous while radiotherapists tended to think the reverse. Mr Buyse has carried out an overview of studies examining whether radiotherapy is of any value in rectal cancer, and the answer seems to be that there may possibly be an improvement of about 5%, but it is not statistically significant. We are just not getting enough patients entered into trials to answer these important questions. Somehow the numbers entering trials have to be increased and trials become a part of routine clinical service. One way of doing this would be to reduce their complexity both in the method of entering patients and in the data to be collected.

*M. Van Glabekke* (Brussels) asked whether more patients were entered into cardiovascular trials because the treatments were very much less toxic. Mr R. Peto felt that one answer to toxicity was to be much more flexible about the treatment protocols in trials. If one wanted to know if radiotherapy was of value or not, then accept a wide range of radiotherapy techniques, so that clinicians could still enter patients, but give a dose in keeping with their individual idea of what is acceptable.

Mr Freedman said that the MRC had not primarily addressed itself to questions in common cancers, where large trials might be both necessary and worthwhile. Perhaps this was something we should be trying to get the MRC to do. Mr R. Peto agreed, but commented that the radiosensitizer questions were good questions, but the trials were of inadequate size to provide answers. Mr Freedman disagreed with this. The glioma trial with 400 patients showed very clearly that the radiosensitizer was not improving survival to the extent that had been expected. The trials of misonidazole were also conducted in diseases that were not all that common, stage III carcinoma of the cervix and head and neck cancer, both these sites being chosen on scientific grounds because of the ability to monitor local control.

*D. Byar* (National Cancer Institute) said that the experience in the USA was not very different from that reported by Mr Buyse for Europe. In fact the Cancer Therapy Evaluation Branch of the Treatment Division of the NCI has been taking a soul-searching look at their own clinical trial activity, because of criticisms suggesting that very little had been discovered by the clinical trials programmes in proportion to the amount of money that had been spent. The important question is in which situations do you want these large simple trials and in which situations are other approaches to research more appropriate. Perhaps the focus should be on identifying more carefully the questions that are worth pursuing and then concentrating our effort on them.

Mr R. Peto wound up the morning session by saying that trials that have been done in the past have acted as a considerable restraint upon what people are likely to believe. A more positive side has been that some trials, such as those in leukaemia, have helped to show how chemotherapy can be given optimally. Trials also provide organised series of patients for centralised study and discussion. The MRC, EORTC and NCI have put a lot of effort into trials and on the whole those trials have done more good than harm. We need to choose a few really good questions where a moderate difference in survival might well exist, or there is a difference in toxicity and no difference in survival. If you are doing a trial, your responsibility is not only to get as many patients as you can into your own trial, but to be aware of trials addressing the same question in other countries and to foster such trials.

The afternoon session was devoted to lessons to be learnt from the past that could help achieve the necessary numbers, and each speaker reported on his experience in trials in a particular area.

*M. Baum* (London) considered the *breast cancer* trials he had been involved in, the first of which was the CRC (Kings/Cambridge) trial comparing post-operative radiotherapy with a watch policy in early breast cancer. There were four essentials for running a large multicentre trial, a real Hypothesis, an adequate Organisation, many Patients and many Events (HOPE). What we need more than anything else in multicentre trials is hope. He agreed with Richard Peto that the best hypotheses we should test are those which are going to help a clinician to resolve his uncertainties in everyday practice.

When the Kings/Cambridge trial was being planned nearly 20 years ago, the statistical advice given was that if they wanted to stand a reasonable chance (9 in 10) of detecting a difference that would be clinically important (about 7%), they must recruit 2,000 patients. It was realised that to get these numbers, a large number of ordinary District Hospitals must be involved. This set the ethos for the group which has been maintained ever since, that one has to have large numbers and to go where the patients are, to the District General Hospitals. One therefore has to have protocols that are pragmatic in design and which can be conducted in a busy DGH practice. His group had worked very hard over the years to simplify protocols and they had now managed to get a protocol onto credit-card size.

Out of the original group developed the CRC Trials Centre at the Rayne Institute. It is essential to have a proper professional organisation with trial coordinators, computing facilities, programmers, office and secretarial staff. In Professor Baum's view, their secret weapon had been their Senior Trials Coordinator, Mrs Joan Houghton, and he wanted to stress that such people were absolutely vital to the success of multicentre clinical trials, and that it was essential for them to have a proper career structure. Liaison with participating centres involves frequent visits to those centres and these visits, together with annual or biannual general meetings, all encourage clinicians to join in trials.

Prof. Baum's group had also over the years made a great effort towards simplification of data collection sheets. Once a patient was randomised, clinicians were supplied with a series of adhesive labels which identified the patient (not by name) and which could be affixed to tear-off pre-addressed cards, on which all necessary follow-up information could be entered.

Regular surveys of clinical practice throughout the country are carried out to help identify what are the current relevant questions which should be addressed by clinical trials of cancer of the breast. A questionnaire in 1980 showed that there was considerable uncertainty about the use of adjuvant systemic therapy in early breast cancer and it would be reasonable to launch a trial with a control arm which had no such adjuvant therapy. In 1983 a questionnaire identified the uncertainty over local excision versus simple mastectomy.

However, the resulting two trials, fared very differently. The adjuvant trial accrued more than 2,000 patients in three and a half years without any problem, while the breast conservation trial which addressed a clinically most relevant question had to be aborted after two and a half years with less than 200 patients. The reason for its failure was an ethical one. The biggest problem now facing trial organisers is the issue of informed consent. We have to persuade clinicians who are frightened by the procedure of informed consent that this is an issue which concerns patients out of trials just as much as those in trials.

*R. Peto* (Oxford) gave the second paper in the afternoon, his subject being a current MRC trial in *prostate cancer*. The median age of patients is 75, and many patients appear to do quite well if left untreated, or at least not treated until their disease causes troublesome symptoms. The question then is whether one should treat immediately at diagnosis or whether one should defer treatment until some evidence emerges that treatment is clearly needed. The best trial that had so far been done in prostate cancer recruited 2,000 patients throughout the USA and showed a significant reduction in mortality from prostate cancer in the immediately treated group, but the difference in overall mortality was not very different. This result was suggestive, but the possible benefits of immediate treatment were not accepted by the British medical profession. Using the USA trial to get an estimate of the possible effect to be looked for, one could calculate that about 2,000 patients were needed for a 90% power. About 50 centres had already started to randomise when there was criticism both in the press and in Parliament that patients were being randomised without their full consent. The trial organisers had said that clinicians should invite patients to enter into the trial in a way that was appropriate to the advice from their local ethical committee, and to give information that was designed with the best interests of the patient at heart. Only a few hundred patients have been entered so far, and it seems that the ethical problem may be one of the main reasons. The trial was simple, and certainly addressed a question of substantial interest to the profession. Mr Peto felt that ethical problems could be a major obstacle to the successful conduct of clinical trials.

*R. Collins* opened the discussion by saying that in one acute myocardial infarction trial (ISIS–2), in which 12,000 patients have been randomised from 400 hospitals, the 50 hospitals in the USA are randomising much slower and have only entered a few hundred patients. This may well be because everywhere except in the USA there is either no consent form or a simple information sheet, whereas in the USA, the lawyers decided that it was necessary to have a three-page closely spaced informed consent form, which the patient is expected to read just after a heart attack, often when he's just been given diamorphine. The form can take an hour to go through properly with a patient and this would appear to have been a major obstacle to randomisation in the USA.

Mr Peto referred back to the principles laid down by Bradford Hill about 30 years ago, and his excellent discussion of informed consent. There are many circumstances in which informed consent before randomisation is not appropriate and the prostate trial is one. To stress to people that they have cancer, that you can't get rid of that cancer, that it may kill them and that you are uncertain of the best treatment is just inhumane. Doctors will answer questions if they are asked them, but they should not have to impose miserable depressing information on patients if it seems humanely inappropriate to do so. What individual people need is different, and the growing version of informed consent that is now becoming standard unfortunately takes no account of these differences. Professor Baum had often said in this context that there seems to be a double standard: patients are supposed for ethical reasons to be told things in clinical trials which one would never dream of telling them in ordinary clinical practice. This double standard is an obstacle to trials and often results in inhumane treatment of the patients in trials.

*S. Pocock* (London) asked if Mr Peto was arguing that in the prostate trial there should be no consent or was he arguing for flexible consent. Mr Peto replied that clinicians should be able to decide what is appropriate for individual patients. He quoted Dr Warlow's trial, where clinicians could either explain the trial before randomisation or they could ring up for the randomisation, and then explain this treatment procedure to the patient. For example, if the patient was allocated to surgery, he would be told that a study was being undertaken to see if surgery would improve the treatment of his condition, he would be told what the treatment and follow-up would involve, and then asked if he would be willing to enter the study. Dr Pocock said this was basically the procedure suggested by Zelen, namely that the patient should be randomised and his consent then obtained to the treatment he has been allocated.

*L. Freedman* (Cambridge) gave the next paper on the experience gained with *rectal cancer* trials. The first MRC rectal cancer trial stopped entry in August 1978, with 850 patients entered at a rate of about 250 per year. The trial compared surgery immediately with pre-operative radiotherapy of 500 cGy in 1 fraction or pre-operative radiotherapy of 2,000 cGy in 10 fractions. At the time of stopping the trial, the analysis showed no apparent difference between the groups in terms of survival, etc., but did show the effects of some prognostic factors. Patients with mobile tumours pre-operatively had a 48% survival at 5 years compared with 29% for those with tethered tumours. The post-operative Dukes' classification was also important; Dukes' A 70% compared with 36% for Dukes' B and C.

Having finished that study, there was debate about whether there should be a larger dose of radiotherapy used, whether radiotherapy should be given pre-operatively or post-operatively, what might be the possible role of chemotherapy, and what the long-term results of the present study might show and what therefore should be the appropriate control group in the second study. It took three years before a new trial was started. This was confined to tethered tumours only and compared immediate surgery with a higher dose of pre-operative radiotherapy, 4,000 cGy in 20 fractions. The predicted entry rate was 100 patients per year but only 40 patients per year have been achieved.

A study for mobile tumours, Dukes' B or C classification, was not started until March 1984. This trial compared immediately surgery with surgery plus post-operative radiotherapy, and was expected to have an entry of 150 patients per year. The entry achieved has been 100 per year.

There were four possible reasons why the rate of patient entry was lower than expected. Firstly, the three-year gap between the end of the first trial and the beginning of the next obviously didn't help. Secondly, since no improvement with pre-operative radiotherapy had been demonstrated in the first trial, some surgeons were put off the idea of using pre-operative radiotherapy again. Also, a third reason, the longer period, 4 weeks, of pre-operative radiotherapy was unpopular with surgeons. The fourth reason was uncertainty whether it was sensible to sub-group patients into different trials. The prognostic difference between mobile and tethered tumours did not necessarily mean that radiotherapy would be more beneficial in one sub-group than in the other.

Professor J. Peto strongly supported this last point. An exactly similar mistake had, in his view, been made recently in the MRC childhood leukaemia trials, where less intensive treatment was being given to the minority of patients with the better prognosis and, not randomised, but only giving the most intensive treatment, with two lots of intensification, to patients with the worse prognosis. This presupposes knowledge which they do not have and now will not obtain from the study.

The next speaker was *C. Warlow* (Oxford – but just moved to Edinburgh) who spoke about his experience of trials in *neurology*. In his specialty there were plenty of burning questions: in the 1970's whether aspirin prevented stroke, in the future the question of radiotherapy treatment of gliomas, and at the moment whether a simple drug treatment, Selegiline given once daily, can prevent or at least reduce the development, or greater development of Parkinson's disease.

The next requirement for large trials is to maximise the number of collaborators. This will increase patient entry and will also make the trial results more able to be generalised to wider clinical practice. There is a problem in neurology in that there are only about 200 neurologists in the UK. The way to go is to extend into Europe where there are a few thousand neurologists who have superb facilities and the time to go to meetings and fill in protocols.

The grey area of uncertainty in clinicians' approaches needs to be exploited. In the European trial on carotid surgery for the prevention of stroke, clinicians differed as to what degree of stenosis would make surgery absolutely essential, and what was so minor that surgery would be unethical. Their grey areas of uncertainty in between, where they were willing to randomise patients, therefore covered very different ranges of stenosis, but nevertheless their patients could be combined in the same trial, and no clinician had to be forced into procedures that he felt unhappy about.

Prof. J. Peto asked what concrete change of policy did people think the MRC and CRC could make which would be beneficial in getting more patients entered into trials. One suggestion he had made was that clinicians should only get support for their research if their patients were being entered into trials or were contributing to a central data bank. Professor Baum felt that financial constraints were not now so much of a problem as the ethical issues, although he certainly thought that the MRC and the CRC should stop funding small trials and concentrate their resources on large ones. Professor Armitage asked if, in fact the MRC did support many small trials.

Mr Freedman thought that the MRC could change its policy in the way that trials are set up. At the moment there is not a conscious effort to define important questions in common cancers. Professor J. Peto emphasised this point by stating that a collaborative group should be established to study a disease rather than just to run a trial. Mr Freedman said that the MRC did in fact set up Working Parties in particular diseases, but their membership was limited. A Working Party was a small group of people who discussed trials and other studies in that particular disease, but participants in those trials or studies would not necessarily be on the Working Party, and might not therefore be involved in discussion about the whole range of studies in that disease. Professor Peto said that he had served on several MRC Working Parties and he did not feel that they did study a disease in the way that he had suggested. Mr Freedman felt that it was a problem of some particular Working Parties rather than the way in which they were set up by the MRC. Professor Baum then mentioned the Breast Cancer Trials Coordinating Committee funded by the MRC and the CRC. This had played an important role in clarifying the important questions in breast cancer. Similar Coordinating Committees had already been started in colorectal cancer and lung cancer. Prof. Armitage said that one shouldn't forget that as long ago as the 1950s the MRC took the initiative in setting up Working Parties to study the possibilities of running clinical trials in various cancer sites.

*Dr Aitken* (MRC Head Office) explained that the Council acknowledged the need for large multicentre trials. In order to achieve adequate numbers of patients for cancer trials the Cancer Therapy Committee was already undertaking some joint studies with other groups such as the EORTC, and collaborative links with the UK cancer charities were being developed. However he emphasised that, particularly for the less common cancers, survival was not the only suitable endpoint. It remained appropriate for the Council's Working Parties to promote smaller trials in these diseases.

*R. Collins* (Oxford) gave the last paper in the afternoon session. His subject was *Acute Myocardial Infarction* trials. These trials had been designed to minimise work in order to maximise trial size. The design was simple: testing practical treatments, with only a one-page discharge form and follow-up mainly through government records in those centres where such records existed. The first study, ISIS 1, was a study of beta-blockade given early in acute myocardial infarction. The study, involving 250 hospitals worldwide and 16,000 randomised patients, demonstrated a reduction in hospital mortality of 15%.

During the $3\frac{1}{2}$ years of recruitment into ISIS 1, the ISIS 2 trial was being planned. This study aimed to test the effect of IV streptokinase on mortality after acute myocardial infarction. First, an overview was made of all trials that had been carried out in the past. The generally held view was that streptokinase had no beneficial effect on mortality, and that it might possibly be harmful since it reduced blood clotting and might therefore increase the risk of haemorrhage and stroke. The overview showed a clearly significant reduction in mortality; typically the risk of death in the treated group was reduced by about 20% with 95% confidence interval from about 10% to 30%. Nevertheless it was clear from the sales of streptokinase that it was not being commonly used.

A similar overview of the randomised trials of aspirin in unstable angina (which is a related condition to acute MI), showed that death or re-infarction was 12% in the control group compared with 7.5% in the aspirin treated group.

A $2 \times 2$ factorial design is therefore used. The treatments tested are very simple: one involves a rapid high-dose infusion of streptokinase given intravenously over one hour, or placebo, and the second involves oral aspirin for a period of one month. It should therefore be possible to assess the effect of streptokinase, the effects of oral aspirin, and to find out whether there is any synergistic effect between the two. It is hoped that the target of about 20,000 patients will have been achieved by the end of 1987.

*Dr Kelly* (Birmingham) said that what she found depressing about the cancer trials was not only the small numbers but also the very low proportion of patients entering such trials. What proportion of all possibly eligible patients were in the cardiovascular trials? Dr Collins said that it was still only a few per cent. Dr Kelly said that rather than stopping funding for inadequately sized trials, it seemed more necessary to recruit the doctors who are not applying for funds and are not entering patients for trials.

Mr R. Peto pointed out that there must be about a million new cancer patients a year in Europe. It should therefore be possible to recruit large numbers as in the cardiovascular trials, if the right questions are asked. Dr Kelly said that cancer is different in that it is many diseases, not one, and that often cytotoxic drugs are used which may raise problems in the smaller centres.

Professor Armitage then closed the meeting with a summary of what he felt had been the main points to emerge during the day's proceedings.